# Diversity of Raft-Like Domains in Late Endosomes

**DOI:** 10.1371/journal.pone.0000391

**Published:** 2007-04-25

**Authors:** Komla Sobo, Julien Chevallier, Robert G. Parton, Jean Gruenberg, F. Gisou van der Goot

**Affiliations:** 1 Department of Microbiology and Molecular Medicine, University of Geneva, Geneva, Switzerland; 2 Department of Biochemistry, University of Geneva, Geneva, Switzerland; 3 Institute for Molecular Bioscience, Centre for Microscopy and Microanalysis, and School of Biomedical Sciences, The University of Queensland, Brisbane, Australia; 4 Ecole Polytechnique Fédérale de Lausanne, SV-AI extension, Lausanne, Switzerland; Duke University Medical Center, United States of America

## Abstract

**Background:**

Late endosomes, the last sorting station in the endocytic pathway before lysosomes, are pleiomorphic organelles composed of tubular elements as well as vesicular regions with a characteristic multivesicular appearance, which play a crucial role in intracellular trafficking. Here, we have investigated whether, in addition to these morphologically distinguishable regions, late endosomal membranes are additionally sub-compartmentalized into membrane microdomains.

**Methodology/Principal Findings:**

Using sub-organellar fractionation techniques, both with and without detergents, combined with electron microscopy, we found that both the limiting membrane of the organel and the intraluminal vesicles contain raft-type membrane domains. Interestingly, these differentially localized domains vary in protein composition and physico-chemical properties.

**Conclusions/Significance:**

In addition to the multivesicular organization, we find that late endosomes contain cholesterol rich microdomains both on their limiting membrane and their intraluminal vesicles that differ in composition and properties. Implications of these findings for late endosomal functions are discussed.

## Introduction

The general view of the cellular plasma membrane has evolved, over the last 20 years, from that of a homogeneous arrangement of lipids with embedded proteins towards that of a mosaic of microdomains, each having a specific lipid and protein composition [1]. Some are morphologically distinguishable, such as clathrin coated pits and caveolae [2], whereas others, such as lipid rafts are apparently featureless regions of the plasma membrane [3], [4]. Assembly of lipid rafts involves not only lateral aggregation of long and saturated acyl chains (glycosphingolipids, phospholipids) in combination with cholesterol [1], [4], [5] but also protein-protein interactions [6] and protein-lipid interactions. This specific lipid environment would then attract certain proteins with high lipid raft partitioning coefficient, such as doubly acylated *src* like kinases or some palmitoylated transmembrane proteins [7], [8], [9], [10]. In addition, modification of lipid raft composition can occur either by changes in the environment or the physiological state of the cell [11] or by the binding of ligands to receptors [12], [13]. Importantly, although a single name is used, rafts are likely to represent a heterogeneous group of domains [1], [14].

Lipid rafts have mostly been studied at the plasma membrane due to their accessibility from the outside of the cell– for microscopy and biophysical studies [6], [15], [16]– and to their role in signaling [12], [13], [17] and endocytosis [18], [19], [20]. Characterization of rafts has also been extensively based on their resistance to detergent solubilization, although this widely used biochemical readout has inherent limitations [21], [22], [23], [24], [25]. Nevertheless, the analysis of detergent resistant membranes (DRMs) remains a useful tool [23] in particular in comparative studies.

In addition to the plasma membrane, many intracellular organelles appear to contain raft-like domains [26], [27], [28], [29], [30]. The endoplasmic reticulum was initially thought to be devoid of cholesterol dependent DRMs because of its low cholesterol content. Several recent studies have however reported their existence [31], [32], [33]. Due to the increase in cholesterol and sphingolipids along the secretory pathway, raft-like domains are thought to become more abundant in the Golgi and more specifically the trans-Golgi network [30], [34], [35]. Raft-like domains are also present in the endocytic pathway, as highlighted by studies on the trafficking of GPI-anchored proteins [18], [29], flotillins [36], toxins and viruses [37]. Occurrence of rafts in the endocytic pathway is probably the combined result of *de novo* assembly and engulfment from the plasma membrane. Endocytosis of raft-like domains can indeed occur both via clathrin-dependent [38], [39] and independent-pathways [18], [19], [36], [40].

Having previously documented the occurrence of DRMs in late endosomes [29], we have characterized these raft-like domains in more detail using morphological approaches and subcellular fractionation followed by sub-organellar fractionation. We show that limiting and internal membranes of this multivesicular compartment [41], [42] both contain raft-like membranes but that these domains differ in their physico-chemical properties and protein composition.

## Materials and Methods

### Cell culture and reagents

Monolayer of baby hamster kidney (BHK), and C2C12 cells were grown and maintained as described by [43], [44], [45]. Aerolysin was purified and labeled as previously described [29], [46]. Our rabbit anti-flotllin-1 polyclonal antibody was previously described [29], anti-NPC1 was from Dr. E. Ikonen (National Public Health Institute, Helsinki, Finland) and anti-MLN64 from Dr J. F. Strauss (University of Pennsylvania, School of Medicine, Philadelphia, USA).

### Subcellular fractionation

Late endosomal fraction was prepared as described [29], [47]. Briefly, BHK cells were harvested and homogenized, a post-nuclear supernatant was prepared and adjusted to 40.6% sucrose, 3 mM imidazole, pH 7.4, loaded at the bottom of an SW41 tube, and overlaid sequentially with 35 and 25% sucrose solutions in 3 mM imidazole, pH 7.4, and then homogenization buffer (HB ; 8.5% sucrose, 3 mM imidazole, pH 7.4). The gradient was centrifuged for 90 min at 35 000 rpm. Early and late endosomal fractions were collected at the 35/25% and 25%/HB interfaces respectively.

### Isolation of DRMs from late endosomal fractions

DRMs were prepared from late endosomes as described [29]. Briefly late endosomes were diluted four times, sedimented by centrifugation (TLS.55 Beckman rotor, 30 min, 55 000 r.p.m.) and resuspended in 200 μl of lysis buffer (25 mM Tris-HCl pH 7.4, 150 mM NaCl, 5 mM EDTA) in the presence of Complete, a cocktail of protease inhibitors (Roche) and 1% Triton X-100. After 20 min of incubation at 4°C, the lysat was adjusted to 40% OptiPrep (Nycodenz), overlaid with a 30% and 0% OptiPrep cushions and centrifuged for 2 h centrifugation at 55 000 rpm (4°C) using a TLS.55 rotor. Six fractions were collected from the top and precipitated with 6% trichloroacetic acid in the presence of sodium deoxycholate as a carrier.

### Sub-fractionation of late endosomes

Late endosomes were described as above and submitted to 5 sequential freezing in liquid nitrogen and thawing at 37°C cycles in order to mechanically disrupt the compartment. Suspension containing broken late endosomes was then centrifuged for 40 minutes at 70000 rpm in a TLA 100.3 rotor. The pellet was resuspended in 500 µl of 40% sucrose in 3 mM imidazole, pH 7.4 loaded at the bottom of a SW40 tube and overlaid with a linear 8–40% sucrose gradient in the same buffer, and centrifuged at 4°C in the SW40 rotor for 16 h at 35000 rpm. Fractions (1 ml each) were collected from the top of the gradient.

### Phospholipid and cholesterol analysis

Lipids were extracted form membrane fractions using CHCl3/MeOH and then separated by two-dimensional thin layer chromatography (TLC) for phospholipids analysis [48], [49]. The first dimension was run with chloroform, methanol, 32% ammonia (65∶35∶5, v/v) and the second with chloroform/acetone/methanol/acetic acid/water (50∶20∶10∶12.5∶5), v/v). Phospholipids were revealed by burning the TLC plate at 160°C after immersion in 1.5 mM cupric acetate-8% H_3_PO_4_ solution. For cholesterol analysis, lipids were extracted as above, analyzed on a one-dimensional TLC in heptane/ethylether/acetic acid (18∶6∶2, v/v) and stained with copper. Both cholesterol and phospholipids were quantified by densitometry using the ScanAnalysis software.

### Immunoblotting, aerolysin overlays and protein quantification

Proteins were separated by SDS-PAGE using 12.5% acrylamide gels unless stated otherwise and transferred onto a nitrocellulose membrane. Western blots were revealed with SuperSignal Chemiluminescence (Pierce). Aerolysin overlays were performed as described [50]. Protein contents of cellular fractions were determined using bicinchoninic acid (BCA, Pierce).

### Electron microscopy

C2C12 cells were fixed in 8% paraformaldehyde and processed for frozen sectioning. Sections were labeled with affinity purified antibodies to flotillin-1 [28] followed by protein A-gold and then with aerolysin-biotin and anti-biotin-gold, as described previously [29].

## Results

### Lipid composition of late endosomal DRMs

**Figure 1:**
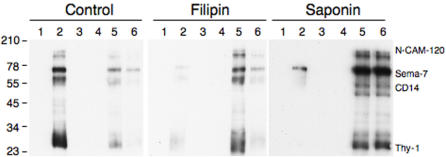
Late endosomal DRMs are sensitive to cholesterol affecting reagents. Late endosomes were prepared from BHK cells using a sucrose step gradient, treated or not with either filipin (1μg/ml for 1 h at 37°C) or saponin (0.4% for 1 h at 4°C) and then submitted to solubilization in 1% Triton X-100 at 4°C. The lysat was submitted to an OptiPrep flotation gradient and 6 fractions of 400 µl were collected. Each fraction was analyzed by SDS-PAGE followed by an aerolysin overlay to identify the GPI-anchored proteins of BKH cells: N-CAM-120, semaphorin-7 (Sema-7), CD14 and Thy-1.

We have previously shown that detergent resistant membranes (DRMs) can be isolated from late endosomes [29] purified from baby hamster kidney (BHK) cells using a well-established subcellular fractionation protocol [29], [47]. These DRMs were found to contain well-characterized raft marker proteins such as GPI-anchored proteins and flotillin-1 (for references concerning these markers see [8], [20], [51], [52]) but were devoid of the transmembrane glycoprotein lamp1 and the lipid anchored GTPase Rab7 [29]. It is important to note that since detergent solubilization was performed on a purified organelle obtained in a relatively low abundance, the detergent to protein ratio used was five to ten times higher, for technical reasons, than that routinely used by us and other on whole cell extracts. Thus the obtained membranes are highly detergent resistant. To test whether late endosomal DRMs are sensitive to cholesterol affecting drugs, an important criterion for being a raft-like domain [4], we treated late endosomes with either the cholesterol clustering agent saponin [53], [54] or the cholesterol binding compound filipin [38]. We did not perform cholesterol extractions using ß-methyl-cyclodextrin, a drug commonly used to disrupt rafts [55], since we have previously shown that on BHK cells this treatment does not to lead the release of GPI-anchored proteins from DRMs [54]. BHK cells contain 4 major GPI-anchored proteins, N-CAM-140, semaphorin-7, CD14 and Thy-1, which can be detected by overlay using the GPI-specific bacterial toxin aerolysin [29]. As shown in Fig. 1, whereas GPI-anchored proteins are abundant in the DRM fraction (fraction 2) of untreated late endosomes, treatment of the purified organelle with either saponin or filipin prior to Triton X-100 solubilization, led to the redistribution of these proteins to the high density detergent soluble fractions on these Optiprep gradients.

**Figure 2:**
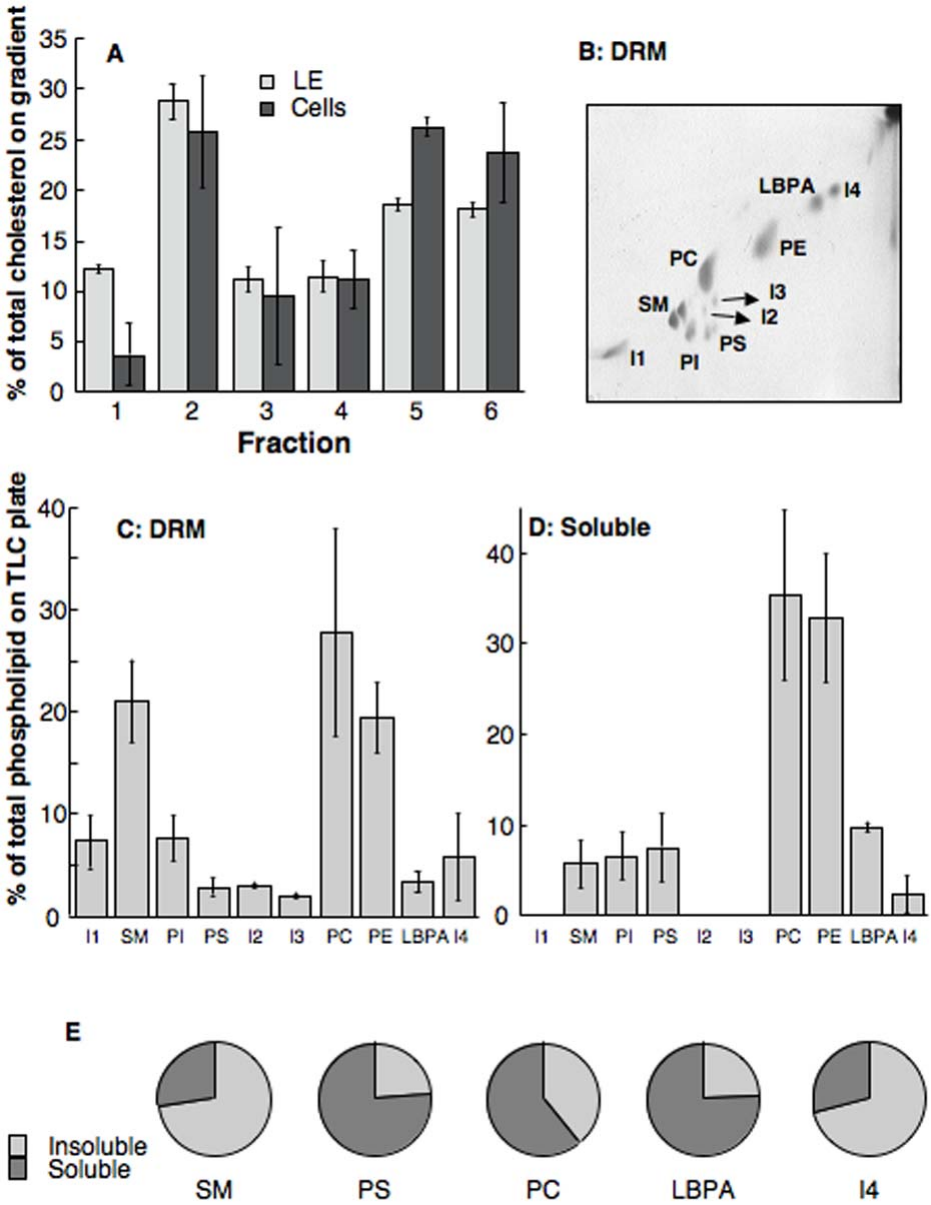
Lipid analysis of late endosomal Triton X-100 resistant membranes. Late endosomes were prepared from BHK cells, submitted to Triton X-100 solubilization and Optiprep gradient analysis as in Fig. 1. Lipids were extracted and analyzed by 1D (A) or 2D TLC (B–E). A: The cholesterol content of each fraction was determined by 1D TLC followed by densitometric analysis. For comparison, total BHK cells were also submitted to Triton solubilization, Optiprep flotation gradients and cholesterol analysis. Error bars represent the standard deviation (n = 3). B: After pooling fractions 1 and 2 from the top of the Optiprep gradient (corresponding to the DRMs), lipids were extracted and analyzed by 2D TLC. I: unidentified lipid, PI: phosphatidylinositol, PS: phosphatidylserine, SM: sphingomyelins, PE: phosphatidylethanolamine, PC: phosphatidylcholine, LBPA: Lysobiphosphatidic acid. C–D: The amount of each phospholipid in fractions 1+2 (C) and fraction 6 (D) were quantified by densitometry. Each phospholipid is expressed as percentage of the total amount of phospholipids on the TLC plate. Error bars represent the standard deviation (n = 3). E: In order to evaluate the distribution of SM, PS, PC, LBPA and I4 through out the Optiprep gradient, the content of these lipids in fractions 1+2 and in fraction 5+6 was determined. For each lipid, the distribution between these two pools was plotted.

These observations indicate that late endosomal DRMs fulfill the criterion of being cholesterol dependent. We next investigated the distribution of cholesterol itself in these DRM fractions. As shown in Fig. 2A, DRMs from late endosomes contain approximately 40% of the total cholesterol content of compartment (Fractions 1 and 2) as determined by thin layer chromatography (TLC), with<40% in soluble membranes (fractions 5 and 6, note that only fraction 6 is fully soluble since fraction 5 already contains the first interface of the step density gradient between 40 and 35% Optiprep). The percentage of detergent resistant cholesterol was somewhat higher in late endosomes than in whole cells (≈30% in DRMs) (Fig. 2A). This observation is all the more significant considering that the high detergent to protein ratio used to isolate late endosomal DRMs when compared to the one used for the isolation of DRMs from total cells.

We next analyzed the lipid composition of the DRMs fractions (fractions 1+2, Fig. 2B and C) in comparison to that of the detergent soluble fraction 6 (Fig. 2D). Lipids were extracted and the relative proportions of phosphatidyl choline (PC), phosphatidyl ethanolamine (PE), phosphatidyl inositol (PI), phosphatidyl serine (PS), sphingomyelin species (SM), lysobisphosphatidic acid (LBPA) were analyzed by 2 dimensional TLC (Fig. 2B). Each spot on the 2D TLC plate was quantified by densitometry and expressed as a percentage of the total intensity on the plate. The three major known phospholipids in DRMs were SM, PC and PE, but, interestingly, 3 unknown lipids (termed I1 to I3) were detected almost exclusively in the DRMs and one was significantly DRM-enriched (I4). By contrast, the unusual late endosomal lipid LBPA [48], although detected in DRMs, was significantly enriched in the soluble fraction. When plotting relative amounts of several phospholipid species in DRM fractions 1+2 vs. fractions 5+6, SM and I4 were mainly present in the insoluble fractions as opposed to LBPA and PS (Fig. 2E). Other lipids such as PC (Fig. 2E) and PI (not shown) were more evenly distributed.

Taken together, these observations show that DRMs from late endosomes share important properties with plasma membrane raft domains: they are rich in cholesterol and sphingomyelin and are sensitive to cholesterol affecting drugs, they contain raft marker proteins such as GPI-anchored proteins and flotillin-1. In addition they are enriched in 4 intriguing yet uncharacterized phospholipids.

### Differential solubilization of DRM associated proteins

**Figure 3:**
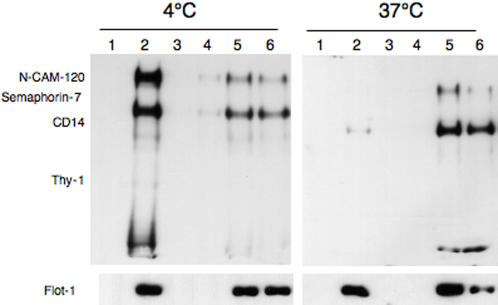
Detection of two types of late endosomal DRMs. Late endosomes from BHK cells were submitted to solubilization in 1% Triton X-100 either at 4°C or at 37°C. The lysat was subsequently analyzed on an Optiprep gradient and 6 fractions were collected from the top. The total of each fraction was submitted to SDS-PAGE and Western blotting to detect flotillin-1 or to an aerolysin overlay to reveal GPI-anchored proteins.

Despite the rather harsh solubilization step (higher detergent to protein ratio than for the preparation of DRMs from whole cells), DRMs isolated from late endosomes contained 40% of the total organellar cholesterol. We therefore wondered whether these domains were particularly resistant to solubilization and therefore performed the solubilization at 37°C. This treatment led to the solubilization of GPI-anchored proteins, but interestingly not to that of flotillin-1 (Fig. 3), suggestive of a differential distribution. Whereas, GPI-anchored proteins and flotillin-1 could be part of the same domain, one being on the periphery and the other in the center, as proposed for prion protein and thy-1 [56], [57], they could also reside on spatially segregated domains. Since late endosomes contain internal vesicles, one attractive possibility is that GPI-anchored proteins and flotillin-1 differentially distribute to the internal and limiting membranes of the organelle.

### Distribution of raft-marker proteins to internal and limiting membranes of late endosomes

**Figure 4:**
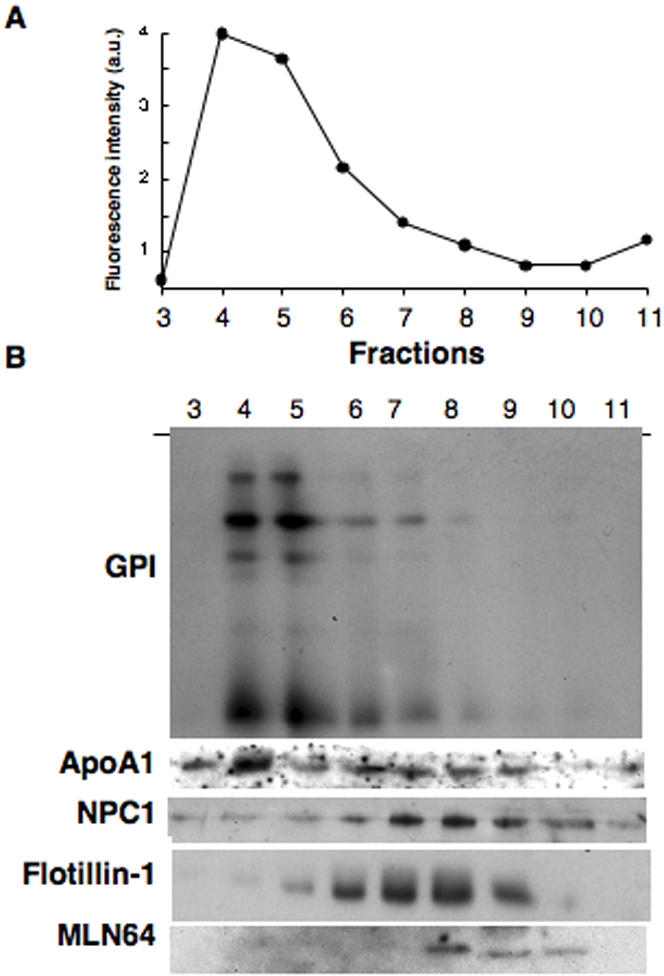
Distribution of lipid metabolism-related proteins and raft markers in late endosomes. Late endosomes were purified from BHK cells and submitted to sub-organellar fractionation after breaking the organelle by cycles of freezing and thawing followed by sucrose density gradients. 12 fractions were collected from the top and analyzed for the presence of LBPA using an ELISA assay (A) or by SDS-PAGE followed by Western blotting for the presence NPC1, MLN64, flotillin-1 and ApoE (B). GPI-anchored proteins were detected by aerolysin overlay (B).

We have previously shown that the internal membranes of late endosomes, which contain high amounts of LBPA [48], can be separated from the limiting membrane, after gentle mechanical disruption (by freeze thawing) of the organelle, followed by a continuous sucrose gradient [49]. Using this sub-organellar fractionation protocol, we analyzed the distribution of flotillin-1 and GPI-anchored proteins as well as that of three other proteins involved in cholesterol metabolism: ApoA1– an LDL apoprotein, MLN64 – a late endosomal steroidogenic acute regulatory protein (StAR) domain containing protein involved in sterol trafficking [58] and NPC1 – the Niemann Pick type C 1 protein involved in lipid trafficking [59]. GPI-anchored proteins co-fractionated with LBPA (Fig 4A), which was quantified by ELISA [49], and were mainly found in fractions 4 and 5 (Fig. 4B), indicating that these contained predominantly the intralumenal membranes of late endosomes. As expected, ApoA1, originating from internalized LDL particles, was also concentrated in fraction 4. In contrast, flotillin-1, MLN64 and NPC1 were all found in fractions 6 to 9 which also contain the limiting membrane marker Lamp1 [49]. These findings are in good agreement with electron microscopy studies in which MLN64 was found to be restricted to the limiting membrane of late endosomes [60]. These data altogether indicate that MLN64 and NPC1, which are both involved in sterol trafficking, localize to the limiting membrane.

**Figure 5:**
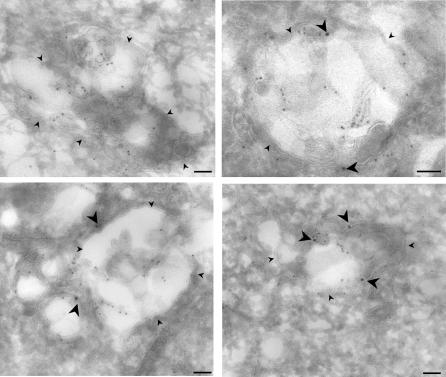
Immunoelectron microscopic localization of flotillin-1 and GPI-anchored proteins on multivesicular endosomes. Cultured C2C12 cells were fixed in paraformaldehyde and processed for frozen sectioning. Sections were labeled with antibodies to flotillin-1 and 15 nm protein A-gold and then overlaid with aerolysin-biotin followed by 10 nm anti-biotin-gold. Aerolysin labeling for GPI-anchored proteins is mainly within the internal membranes of the late endosomes. In contrast, flotillin-1 labeling (large arrowheads) is predominantly associated with the limiting membrane (small arrowheads). Bars, 100 nm.

The segregation between GPI-anchored proteins and flotillin-1 was confirmed by electron microscopy using C2C12 cells (Fig. 5, the flotillin-1 antibodies showed negligible labeling by immunoelectron microscopy on BHK cells). For quantifications, frozen sections were double labeled for GPI-anchored proteins (using aerolysin) and flotillin-1. Well-preserved multivesicular late endosomes were examined at random and gold particles (n = 450) were assigned to the limiting membrane or to internal membranes. For 85% of late endosomes, flotillin-1 labeling was higher on limiting membranes with a ratio of 4.8 to 1. On 15% of late endosomes, flotillin-1 was however more abundant on internal membranes leading to an over all ratio of labeling on limiting vs. internal membranes of 3.1 .to 1. The distribution of GPI-anchored proteins was the reverse with a ratio of limiting to internal membranes of 0.3 to 1 for 80% of late endosomes. Again 20% of late endosomes behaved differently showing a higher GPI labeling on the limiting membrane leading to an over all labeling ratio of 0.44 to 1 of limiting to internal membranes.

Altogether these observations indicate that detergent resistant membrane domains, with different biochemical properties and different protein composition, are found on the limiting and internal membranes of late endosomes, the former being more resistant and containing flotillin-1, and the latter containing GPI-anchored proteins.

## Discussion

It has long been known that late endosomes have a complex morphology with tubular and vesicular regions, which in turn can be multivesicular or multilamellar [41], [42]. These morphological distinct areas, which by themselves define different membrane domains, are likely to be further divided into macro or microdomains. Consistently, Rab9 and Rab7, two late endosomal Rab proteins, occupy distinct domains within late endosomal membranes [61]. Here we have studied the existence of lipid raft-like domains in late endosomes. We used multiple assays, the first of which was the isolation of detergent resistant membranes from the purified organelle. Although this method should be used with care and has its drawbacks [4], [21], [62], it remains powerful in combination with other methods and in comparative studies on the same cell type, complementary approaches which we carried out here.

Our data indicate that late endosomal DRMs are rich in cholesterol (40% of the cholesterol present in the compartment) and in sphingomyelin (70% of that in the organelle), sensitive to cholesterol affecting drugs and contain well-characterized raft-marker proteins, altogether supporting that these DRMs contain raft-like domains [4], [23], [55]. These DRMs also contained some LBPA, a lipid that is confined to late endosomes and abundant in intralumenal membranes. While spurious association of LBPA to DRMs cannot be excluded at this point, it is possible that the unusual nature of this non-hydrolysable lipid confers special properties to these domains, including fusogenic properties [49]. Interestingly, late endosomal DRMs also contained 3 unidentified lipids, I2, I3, and I4, which were not present in the detergent soluble membranes and will be of interest for future studies.

Solubilization of late endosomes at different temperatures revealed differential behavior hinting towards the existence of different raft-like domains within this complex compartment. This hypothesis was supported by sub-organellar fractionation and electron microscopy. More specifically, we found that flotillin-1-positive domains reside on the limiting membrane of late endosomes and are very resistant to detergent solubilization whereas GPI-domains reside on intraluminal vesicles and are more detergent sensitive. Our finding that such raft-like membranes, containing GPI-anchored proteins, are present within intralumenal membranes of these multivesicular endosomes fits nicely with electron microscopy observations using a cholesterol-binding toxin showing that cholesterol is abundant within these lumenal membranes [63]. It has recently been shown that GPI-anchored proteins can be endocytosed from the plasma membrane via a flotillin-1 dependent pathway [36]. Understanding how GPI-anchored proteins and flotillin-1 segregate from one another at later stages of the endocytic pathway will be of great interest. Importantly, both limiting and luminal membranes also contain fluid membranes as illustrated by the detergent sensitivity of lamp1 and LBPA respectively. Thus both limiting and luminal membranes are composed of diverse lipid domains.

It is now well-accepted that the sorting of down regulated signaling receptors into intralumenal membranes mediates their lysosomal targeting and degradation [64]. By contrast, some proteins, like the major glycoprotein Lamp1 [49], [65], the sterol traffic regulator MLN64 [60] and flotillin-1 (this study) remain preferentially on the limiting membrane. In addition, some proteins and receptors can also be sorted into late endosomes, but then recycle back to the limiting membrane, presumably via back-fusion of intralumenal vesicles with the limiting membranes [66] — a process hijacked by some toxins and viruses [67]. It is tempting to speculate that protein and lipid sorting into and out of endosomes may be controlled, at least in part, by differential partitioning into different raft-like membrane domains. In addition, such differences in the protein composition and physico-chemical properties of these two pools of raft domains likely affect their function, which could be altered in lipid storage diseases which have been shown to lead to cholesterol accumulation in late endosomes.
